# National Vaccination Coverage Among Adolescents Aged 13–17 Years — National Immunization Survey-Teen, United States, 2023

**DOI:** 10.15585/mmwr.mm7333a1

**Published:** 2024-08-22

**Authors:** Cassandra Pingali, David Yankey, Michael Chen, Laurie D. Elam-Evans, Lauri E. Markowitz, Carla L. DeSisto, Sarah F. Schillie, Michelle Hughes, Madeleine R. Valier, Shannon Stokley, James A. Singleton

**Affiliations:** ^1^Immunization Services Division, National Center for Immunization and Respiratory Diseases, CDC; ^2^Division of Viral Diseases, National Center for Immunization and Respiratory Diseases, CDC; ^3^Division of Bacterial Diseases, National Center for Immunization and Respiratory Diseases, CDC.

**Summary**

**What is already known about this topic?**

Vaccinations are the best defense to protect persons and communities from serious vaccine-preventable diseases. Three vaccines are routinely recommended for adolescents. The Vaccines for Children (VFC) program provides recommended vaccines at no cost to eligible children and adolescents.

**What is added by this report?**

Overall, vaccination coverage among VFC-eligible adolescents remained stable across recent birth years, except for a decline in human papillomavirus vaccine up-to-date coverage by age 13 years among those born in 2010 compared with those born in 2007.

**What are the implications for public health practice?**

Health care providers should make strong recommendations for all routine vaccines and verify if adolescents, particularly those eligible for the VFC program, are up to date with all recommended vaccines.

## Abstract

Based on safety and efficacy data, vaccinations are the best defense to protect persons and communities from serious vaccine-preventable diseases. The Advisory Committee on Immunization Practices recommends routine vaccination of adolescents aged 11–12 years with three vaccines including tetanus, diphtheria, and acellular pertussis vaccine; quadrivalent meningococcal conjugate vaccine; and human papillomavirus vaccine. CDC analyzed data from the 2023 National Immunization Survey-Teen for 16,658 adolescents aged 13–17 years (born during January 2005–December 2010) to assess vaccination coverage in 2023, recent trends in coverage by birth year, and trends in coverage by eligibility for the Vaccines for Children (VFC) program and birth year. In 2023, coverage with all routine vaccines recommended for adolescents was similar to coverage in 2022. Vaccination coverage among VFC-eligible adolescents was generally stable during the COVID-19 pandemic, except for a decrease in the percentage of VFC-eligible adolescents who were up to date with HPV vaccination by age 13 years among those born in 2010 compared with those born in 2007. Whereas coverage differences were found between VFC-eligible and non–VFC-eligible adolescents before the COVID-19 pandemic, coverage was similar among the most recent birth years in the survey. Providers should make strong recommendations for all routine vaccines and review adolescent vaccination records to verify if adolescents are up to date with all recommended vaccines.

## Introduction

Based on safety and efficacy data, vaccinations are the best defense to protect persons and communities from serious vaccine-preventable diseases. 2024 marks the 30th anniversary of the Vaccines for Children (VFC) program, which provides recommended vaccines at no cost to eligible children and adolescents (*1*). The Advisory Committee on Immunization Practices (ACIP) recommends routine vaccination of children aged 11–12 years with tetanus, diphtheria, and acellular pertussis vaccine (Tdap); quadrivalent meningococcal conjugate vaccine (MenACWY); and human papillomavirus (HPV) vaccine (which may begin at age 9 years). At age 16 years, adolescents should receive a booster dose of MenACWY. In addition, persons aged 16–23 years may receive serogroup B meningococcal vaccine (MenB) on the basis of shared clinical decision-making. Adolescents should also catch up on missed childhood vaccines, stay current with COVID-19 vaccinations,* and receive an annual influenza vaccine^†^ (*2*). A recent publication used National Immunization Survey-Child data to assess trends in vaccination coverage among VFC-eligible children aged 19–35 months (*3*). This new report includes the first assessment of vaccination coverage trends by birth year among adolescents eligible for the VFC program. This report uses 2015–2023 National Immunization Survey-Teen (NIS-Teen) data to assess 1) trends in coverage by eligibility for the VFC program^§^ and birth year, 2) vaccination coverage in 2023 among adolescents aged 13–17 years, and 3) recent trends in coverage by birth year.

## Methods

NIS-Teen is a random–digit-dialed mobile telephone survey^¶^ conducted in the United States to monitor vaccination coverage among adolescents aged 13–17 years.** A household survey is administered to parents or guardians of eligible adolescents to collect information about the adolescent and the household, and to obtain consent to contact the adolescent’s vaccination providers. Once consent is received, a mailed survey is sent to all vaccination providers identified by the parent or guardian to compile the adolescent’s complete vaccination record.

The 2023 NIS-Teen vaccination coverage estimates were derived from provider-reported data for 16,658 adolescents aged 13–17 years^††^ who were born during January 2005–December 2010.^§§^ The household response rate^¶¶^ was 24.4%, and 39.5% of adolescents with completed interviews had adequate provider data.*** Cross-sectional analysis was used to estimate vaccination coverage among adolescents aged 13–17 years in the 2023 survey year compared with the 2022 survey year. Using methodology established in 2021 and 2022 NIS-Teen reports (*4*,*5*), vaccination coverage by birth year was assessed using combined 2015–2023 NIS-Teen data. To assess coverage trends before and after the COVID-19 pandemic began, the 2007–2010 birth years were compared by age using the 2007 birth year as the reference group, because this was the last birth year that consisted of adolescents whose routine vaccinations were not affected by health care disruptions during the pandemic. To assess coverage trends by VFC eligibility, the 2002–2010 birth years were evaluated. Kaplan-Meier techniques were used to account for censoring of vaccination status at age ≥13 years.^†††^ Z-tests were used to compare differences in vaccination coverage by survey year, birth year, and eligibility for the VFC program; differences with p<0.05 were considered statistically significant. Data were weighted^§§§^ and analyses were conducted using SAS-callable SUDAAN (version 11; RTI International). This activity was reviewed by CDC, deemed not research, and was conducted consistent with applicable federal law and CDC policy.^¶¶¶^

## Results

### Recent Trends in Vaccination Coverage by Age 13 Years and by Eligibility for the VFC Program

Coverage with ≥1 Tdap dose, ≥1 MenACWY dose, and ≥1 HPV vaccine dose by age 13 years among adolescents born during 2008–2010 and eligible for the VFC program was similar to coverage among VFC-eligible adolescents born in 2007. However, the percentage of VFC-eligible adolescents who were up to date with HPV vaccination (HPV UTD)**** was 10.3 percentage points lower among adolescents born in 2010 compared with those born in 2007 (Figure) (Table 1).

**FIGURE:**
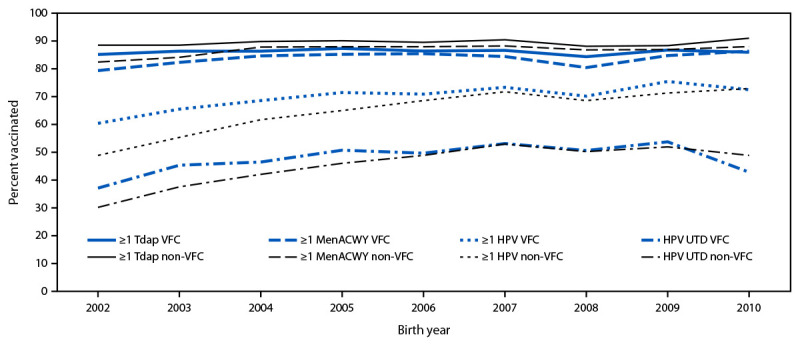
Vaccination coverage,* by age 13 years among adolescents born during 2002–2010,^†^ by Vaccines for Children program eligibility^§^ — National Immunization Survey-Teen, United States, 2015–2023 **Abbreviations:** HPV = human papillomavirus; HPV UTD = up to date with HPV vaccination; MenACWY = quadrivalent meningococcal conjugate vaccine; Tdap = tetanus, diphtheria, and acellular pertussis vaccine; VFC = Vaccines for Children. * At least 1 dose Tdap at age ≥10 years, ≥1 dose MenACWY or meningococcal-unknown type vaccine, and ≥1 dose HPV vaccine (nine-valent, quadrivalent, or bivalent). The routine Advisory Committee on Immunization Practices recommendation for HPV vaccination was made for females in 2006 and for males in 2011. Because HPV vaccination was recommended for males in 2011, coverage for all adolescents was not measured before that year. HPV UTD includes adolescents who received ≥3 doses, and those who received 2 doses when the first HPV vaccine dose was initiated at age <15 years and there was ≥5 months minus 4 days between the first and second dose. ^†^ National Immunization Survey-Teen data during 2015–2023 were combined, and Kaplan-Meier methods were used to calculate cumulative vaccination coverage estimates by age in days, stratified by annual birth year; sample sizes by birth year were 19,931 (2002), 20,085 (2003), 18,908 (2004), 18,242 (2005), 16,564 (2006), 12,633 (2007), 8,346 (2008), 4,990 (2009), and 1,692 (2010). ^§^ VFC-eligible adolescents were defined as meeting one of the following criteria: 1) enrolled in Medicaid or Indian Health Service; 2) uninsured; 3) American Indian or Alaska Native; 4) ever received a vaccination at Indian Health Service–operated centers, Tribal health centers, or urban Indian health care facilities.

**TABLE 1:** Vaccination coverage* among adolescents born during 2007–2010,^†^ by age, and by Vaccines for Children program eligibility^§^ — National Immunization Survey-Teen, United States, 2015–2023

Vaccine	Birth year, % (95% CI)^¶^								
	By age 13 yrs				By age 14 yrs			By age 15 yrs	
	2007	2008	2009	2010	2007	2008	2009	2007	2008
**Overall**									
≥1 dose Tdap	88.7 (87.5–89.9)	86.4 (84.7–88.1)**	87.5 (85.4–89.5)	88.6 (85.1–91.5)	90.5 (89.4–91.5)	88.0 (86.2–89.6)**	88.6 (86.4–90.6)	91.0 (89.9–92.0)	88.5 (86.7–90.2)**
≥1 dose MenACWY	86.6 (85.2–87.9)	84.0 (82.1–85.9)**	85.8 (83.8–87.8)	87.2 (83.4–90.5)	88.9 (87.6–90.1)	86.5 (84.5–88.3)**	86.8 (84.7–88.8)	89.7 (88.4–90.8)	87.2 (85.2–89.0)**
≥1 dose HPV	72.4 (70.8–74.0)	69.2 (67.0–71.4)**	73.2 (70.7–75.5)	72.6 (68.1–76.9)	77.1 (75.5–78.7)	73.5 (71.3–75.8)**	75.6 (73.0–78.1)	79.3 (77.7–80.9)	75.9 (72.8–78.8)**
HPV UTD	52.9 (51.1–54.7)	50.3 (48.1–52.5)	52.6 (49.9–55.5)	45.8 (41.2–50.7)**	60.9 (59.0–62.8)	57.0 (54.6–59.4)**	58.5 (55.3–61.7)	65.2 (63.2–67.2)	60.3 (57.1–63.6)**
**Non–VFC-eligible**									
≥1 dose Tdap	90.4 (89.2–91.5)	88.1 (86.0–90.0)	88.3 (85.6–90.6)	91.0 (88.2–93.4)	91.2 (90.0–92.2)	89.3 (87.2–91.1)	89.0 (86.3–91.4)	91.4 (90.2–92.4)	NA
≥1 dose MenACWY	88.2 (86.6–89.8)	86.8 (84.5–88.9)	86.9 (84.3–89.2)	88.0 (83.9–91.5)	89.3 (87.6–90.8)	88.7 (86.5–90.7)	87.5 (84.9–89.9)	89.7 (88.0–91.2)	88.9 (86.7–90.9)
≥1 dose HPV	71.7 (69.7–73.7)	68.5 (65.7–71.3)	71.2 (68.0–74.3)	72.8 (66.9–78.4)	76.7 (74.7–78.7)	73.9 (71.0–76.8)	73.8 (70.3–77.2)	78.6 (76.5–80.5)	76.7 (72.2–81.0)
HPV UTD	52.8 (50.5–55.0)	50.1 (47.4–52.9)	51.8 (48.4–55.4)	48.7 (42.9–54.9)	61.8 (59.4–64.1)	56.7 (53.7–59.8)**	57.5 (53.6–61.6)	66.5 (64.0–69.0)	60.6 (56.0–65.2)**
**VFC-eligible**									
≥1 dose Tdap	86.6 (84.3–88.7)^††^	84.3 (81.2–87.1)^††^	86.7 (83.2–89.8)	85.9 (79.4–91.2)	89.6 (87.7–91.4)	86.3 (83.3–89.0)	88.1 (84.5–91.3)	90.5 (88.6–92.3)	87.5 (84.5–90.3)
≥1 dose MenACWY	84.4 (82.1–86.6)^††^	80.4 (76.9–83.6)^††^	84.7 (81.3–87.8)	86.3 (79.7–91.7)	88.3 (86.3–90.2)	83.6 (80.1–86.7)**^,††^	86.0 (82.5–89.2)	89.6 (87.6–91.4)	85.0 (81.5–88.1)**
≥1 dose HPV	73.3 (70.7–76.0)	70.1 (66.6–73.5)	75.4 (71.7–79.0)	72.4 (65.4–78.9)	77.6 (75.0–80.1)	72.9 (69.4–76.4)**	77.6 (73.6–81.3)	80.2 (77.6–82.7)	74.5 (70.8–78.1)**
HPV UTD	53.0 (50.1–56.0)	50.5 (46.9–54.2)	53.6 (49.2–58.1)	42.7 (35.6–50.7)**	59.9 (56.8–63.0)	57.4 (53.7–61.3)	59.5 (54.4–64.7)	63.6 (60.3–66.8)	59.8 (55.7–63.9)

**Abbreviations:** HPV = human papillomavirus; HPV UTD = up to date with HPV vaccination; MenACWY = quadrivalent meningococcal conjugate vaccine; NA = not available; Tdap = tetanus, diphtheria, and acellular pertussis vaccine; VFC = Vaccines for Children.

* At least 1 dose Tdap at age ≥10 years; ≥1 dose MenACWY or meningococcal-unknown type vaccine; and ≥1 dose HPV vaccine (nine-valent, quadrivalent, or bivalent). The routine Advisory Committee on Immunization Practices recommendation for HPV vaccination was made for females in 2006 and for males in 2011. Because HPV vaccination was recommended for males in 2011, coverage for all adolescents was not measured before that year. HPV UTD includes adolescents with ≥3 doses, and those with 2 doses when the first HPV vaccine dose was initiated at age <15 years and there was ≥5 months minus 4 days between the first and second dose.

^†^ National Immunization Survey-Teen data during 2015–2023 were combined, and Kaplan-Meier methods were used to calculate cumulative vaccination coverage estimates by age in days, stratified by annual birth year; sample sizes by birth year were 12,633 (2007), 8,346 (2008), 4,990 (2009), and 1,692 (2010).

^§^ VFC-eligible adolescents were defined as meeting one of the following criteria: 1) enrolled in Medicaid or Indian Health Service; 2) uninsured; 3) American Indian or Alaska Native; 4) ever received a vaccination at Indian Health Service–operated centers, Tribal health centers, or urban Indian health care facilities.

^¶^ Estimates with 95% CI >20 might not be reliable. https://www.cdc.gov/vaccines/imz-managers/nis/downloads/NIS-TEEN-PUF22-DUG.pdf

** Statistically significant difference (p<0.05); referent group was the 2007 birth year.

^††^ Statistically significant difference (p<0.05) between non–VFC-eligible adolescents and VFC-eligible adolescents born in the same year; referent group was non–VFC-eligible adolescents.

Among adolescents born during 2003–2008, coverage with ≥1 Tdap and ≥1 MenACWY dose by age 13 years was lower among VFC-eligible adolescents than among non–VFC-eligible adolescents, whereas among those born in 2009 and 2010, coverage was similar among both VFC-eligible and non–VFC-eligible adolescents. Coverage with ≥1 HPV vaccine dose and percentage HPV UTD was higher among VFC-eligible adolescents than among non–VFC-eligible adolescents born during 2002–2005, whereas coverage was similar by VFC eligibility status among those born during 2006–2010 (Figure).

### Routine Vaccination Coverage Among Adolescents Aged 13–17 Years, by Survey Year

In 2023, coverage with all routine vaccines recommended for adolescents was similar to coverage in 2022. Among adolescents aged 13–17 years included in the 2023 survey, 89.0% had received ≥1 Tdap dose,^††††^ 88.4% had received ≥1 MenACWY dose,^§§§§^ 76.8% had received ≥1 HPV vaccine dose,^¶¶¶¶^ and 61.4% were HPV UTD (Table 2) (Supplementary Figure 1, https://stacks.cdc.gov/view/cdc/159388). Among the other vaccines and catch-up vaccines recommended for adolescents, coverage with ≥1 MenB dose***** increased by 3.0 percentage points and coverage with ≥2 hepatitis A vaccine doses^†††††^ increased by 1.9 percentage points in 2023 compared with coverages in 2022.

**TABLE 2:** Estimated vaccination coverage with selected vaccines and doses among adolescents aged 13–17* years, by age at time of interview — National Immunization Survey-Teen, United States, 2023

Vaccine	% (95% CI)^†^							
	Age at interview, yrs						Total	
	13 n = 3,295		14 n = 3,376	15 n = 3,343	16 n = 3,382	17 n = 3,172	2023 n = 16,568	2022 n = 16,043
**Tdap^§^ ≥1 dose**		88.6 (86.1–90.7)	87.1 (84.2–89.5)	90.7 (88.6–92.4)	89.7 (87.5–91.5)	88.9 (86.0–91.2)	**89.0 (87.9–90.0)**	**89.9 (88.9–90.9)**
**MenACWY^¶^**								
≥1 dose		85.1 (82.2–87.6)	86.0 (82.9–88.6)	89.4 (87.2–91.3)**	90.3 (88.2–92.1)**	91.2 (89.0–93.0)**	**88.4 (87.3–89.4)**	**88.6 (87.6–89.6)**
≥2 doses^††^		NA	NA	NA	NA	59.7 (56.2–63.2)	**59.7 (56.2–63.2)**	**60.8 (57.5–63.9)**
**MenB^§§^**								
≥1 dose		NA	NA	NA	NA	32.4 (29.3–35.6)**	**32.4 (29.3–35.6)^¶¶^**	**29.4 (26.5–32.4)**
≥2 doses		NA	NA	NA	NA	12.8 (10.7–15.3)	**12.8 (10.7–15.3)**	**11.9 (10.0–14.1)**
HPV*** vaccine								
**All adolescents**								
≥1 dose		72.2 (69.1–75.2)	76.4 (73.2–79.3)	77.7 (74.5–80.6)**	79.6 (76.7–82.3)**	77.7 (74.4–80.7)**	**76.8 (75.4–78.1)**	**76.0 (74.7–77.3)**
HPV UTD^†††^		49.0 (45.5–52.5)	60.1 (56.7–63.4)**	62.3 (58.4–66.0)**	69.0 (65.7–72.1)**	66.1 (62.4–69.6)**	**61.4 (59.9–63.0)**	**62.6 (61.1–64.0)**
**Females**								
≥1 dose		74.3 (69.8–78.3)	79.0 (74.9–82.6)	77.0 (72.6–80.9)	81.6 (77.4–85.1)**	80.8 (77.1–84.1)**	**78.5 (76.7–80.2)**	**77.8 (75.8–79.6)**
HPV UTD		52.3 (47.2–57.4)	61.9 (57.1–66.5)**	65.2 (60.1–69.9)**	70.4 (65.4–74.9)**	70.1 (65.6–74.1)**	**64.0 (61.9–66.1)**	**64.6 (62.5–66.6)**
**Males**								
≥1 dose		70.2 (65.6–74.5)	73.9 (69.0–78.3)	78.3 (73.6–82.4)**	77.9 (73.6–81.6)**	74.7 (69.3–79.5)	**75.1 (73.0–77.1)**	**74.4 (72.5–76.1)**
HPV UTD		45.7 (41.1–50.4)	58.4 (53.5–63.1)**	59.6 (53.8–65.1)**	67.8 (63.4–71.8)**	62.3 (56.6–67.7)**	**59.0 (56.7–61.2)**	**60.6 (58.6–62.6)**
**MMR ≥2 doses**		93.3 (91.3–94.9)	90.5 (87.9–92.5)	91.5 (88.1–93.9)	90.6 (88.2–92.5)	90.7 (88.1–92.7)	**91.3 (90.2–92.3)**	**91.2 (90.2–92.1)**
**Hepatitis A vaccine ≥2 doses^§§§^**		88.6 (86.2–90.7)	87.9 (85.2–90.2)	86.6 (83.3–89.4)	85.5 (82.8–87.8)	85.8 (83.1–88.1)	**86.9 (85.7–88.0)^¶¶^**	**85.0 (83.8–86.1)**
**Hepatitis B vaccine ≥3 doses**		93.1 (91.4–94.6)	89.9 (87.1–92.1)**	90.6 (87.3–93.1)	89.8 (87.3–91.8)**	91.0 (88.6–92.9)	**90.9 (89.8–91.9)**	**91.2 (90.2–92.1)**
**Varicella**								
History of varicella disease^¶¶¶^		7.1 (5.0–9.9)	6.9 (5.2–9.0)	7.2 (5.6–9.2)	8.1 (6.2–10.4)	7.1 (5.2–9.6)	7.3 (6.4–8.2)	7.0 (6.3–7.8)
**No history of varicella disease**								
≥1 dose vaccine		96.3 (95.2–97.1)	94.1 (91.8–95.8)	94.9 (92.4–96.7)	92.9 (90.7–94.6)**	95.0 (93.3–96.3)	94.6 (93.8–95.4)	94.1 (93.2–94.8)
≥2 doses vaccine		93.2 (91.4–94.6)	89.7 (87.0–91.9)**	91.7 (89.0–93.8)	89.9 (87.6–91.9)**	89.6 (86.8–91.9)**	90.8 (89.8–91.8)	90.8 (89.8–91.8)
History of varicella disease or receipt of ≥2 varicella vaccine doses		93.7 (92.0–95.0)	90.4 (87.9–92.5)**	92.3 (89.8–94.3)	90.7 (88.6–92.5)**	90.4 (87.8–92.5)**	91.5 (90.5–92.4)	91.5 (90.5–92.4)

**Abbreviations:** HPV = human papillomavirus; HPV UTD = up to date with HPV vaccination; MenACWY = quadrivalent meningococcal conjugate vaccine; MenB = serogroup B meningococcal vaccine; MMR = measles, mumps, and rubella vaccine; NA = not applicable; NIS-Teen = National Immunization Survey-Teen; Tdap = tetanus, diphtheria, and acellular pertussis vaccine.

* Adolescents (16,658) in the 2023 NIS-Teen were born during January 2005–December 2010.

**^†^** Estimates with 95% CI >20 might not be reliable. https://www.cdc.gov/vaccines/imz-managers/nis/downloads/NIS-TEEN-PUF22-DUG.pdf

^§^ Includes percentages of persons receiving Tdap vaccine at age ≥10 years.

^¶^ Includes percentages of adolescents receiving MenACWY or an unknown type of meningococcal vaccine.

** Statistically significant difference (p<0.05) in estimated vaccination coverage by age; referent group was adolescents aged 13 years.

^††^ At least 2 doses of MenACWY or unknown type of meningococcal vaccine among adolescents aged 17 years at time of interview and does not include adolescents who received first dose of MenACWY vaccine at age ≥16 years.

^§§^ Calculated only among adolescents who were aged 17 years at time of interview with vaccine administered based on individual clinical decision; ≥2 doses of MenB required correct interval between first and second dose and excluded those with unknown type of meningococcal vaccine.

^¶¶^ Statistically significant difference (p<0.05) compared with 2022 NIS-Teen estimates.

*** HPV vaccine, nine-valent, quadrivalent, or bivalent. For ≥1 dose and HPV UTD measures, percentages are reported among females and males combined (16,568) and for females only (7,857) and males only (8,711).

^†††^ HPV UTD includes adolescents who received ≥3 doses, and those who received 2 doses when the first HPV vaccine dose was initiated at age <15 years and there was ≥5 months minus 4 days between the first and second dose (https://www.cdc.gov/iis/cdsi/). This update to the HPV vaccine recommendation occurred in December 2016.

^§§§^ In July 2020, the Advisory Committee on Immunization Practices revised recommendations for hepatitis A vaccination to include catch-up vaccination for children and adolescents aged 2–18 years who have not previously received hepatitis A vaccine at any age.

^¶¶¶^ By parent or guardian report or provider records.

### Recent Trends in Vaccination Coverage, by Birth Year

Among adolescents born in 2008 (i.e., due for routine adolescent vaccines during the pandemic), coverage by age 13 years with ≥1 Tdap dose was 2.3 percentage points lower, ≥1 MenACWY dose was 2.6 percentage points lower, and ≥1 HPV vaccine dose was 3.2 percentage points lower than among those born in 2007 (i.e., due for routine adolescent vaccines before the pandemic began) (Table 1) (Supplementary Figure 2, https://stacks.cdc.gov/view/cdc/159389). Routine vaccination coverage by age 14 and 15 years among adolescents born in 2008 remained lower than coverage among adolescents born in 2007. Coverage attained by age 13 and 14 years among adolescents born in 2009 was similar to prepandemic coverage levels. Among those born in 2010, coverage by age 13 years was similar to prepandemic coverage levels, except that the percentage of adolescents who were HPV UTD was 7.1 percentage points lower than among those born in 2007.

## Discussion

For three decades, the VFC program has been instrumental in maintaining and progressively improving vaccination coverage among children and adolescents. Approximately 40% of adolescents aged 13–17 years included in the 2023 NIS-Teen data were eligible for the VFC program, highlighting the program’s critical role in achieving high vaccination coverage across the United States (CDC, unpublished data, 2023). The effectiveness of the VFC program in reaching vulnerable and under-resourced communities is demonstrated by the higher HPV vaccination coverage among VFC-eligible adolescents compared with non–VFC-eligible adolescents before the COVID-19 pandemic, and similar coverage by VFC eligibility since the pandemic. The decline in the percentage of VFC-eligible adolescents who are HPV UTD could signal a change in accessibility to vaccination through the VFC program, a change that needs further exploration. This possibility underscores the importance of ongoing efforts to ensure equitable access to vaccination services for all children and adolescents. Significant opportunities to improve vaccination coverage among VFC-eligible adolescents remain, highlighting the need for continued outreach and support to address barriers to vaccination among these populations.

In 2023, with the exception of small increases in vaccination coverage for ≥1 MenB dose and ≥2 hepatitis A doses, coverage with routine vaccines recommended for adolescents was similar to 2022 coverage estimates. For the second consecutive year, HPV vaccination coverage has not increased among adolescents aged 13–17 years.

Lower vaccination coverage among adolescents born in 2008 compared with those born in 2007 was first identified with the 2021 NIS-Teen report (*4*) and has persisted, demonstrating the ongoing impact of disruptions to health care during the COVID-19 pandemic. However, coverage with ≥1 Tdap dose, ≥1 MenACWY dose, and ≥1 HPV vaccine dose among adolescents born in 2009 and 2010 returned to prepandemic levels. In 2021, approximately 25% of U.S. households reported that a child or adolescent had missed or delayed a health care visit because of the pandemic (*6*,*7*). Outreach to parents of adolescents who have yet to return to routine medical care since the pandemic will be critical to verifying that adolescents receive important primary care and vaccinations. In addition, compared with coverage among adolescents born in 2007, HPV UTD coverage among those born in 2010 decreased 7.1 percentage points overall and 10.3 percentage points among VFC-eligible adolescents. HPV vaccination is essential to prevent HPV-attributable cancers (*8*). Although HPV vaccine initiation by birth year has returned to prepandemic levels, further efforts are needed to increase HPV vaccination coverage.

### Limitations

The findings in this report are subject to at least three limitations. First, the household response rate was low, which could introduce selection bias if respondents differed systematically from nonrespondents. Second, coverage estimates stratified by birth year were derived from unequal sample sizes. Sample sizes among younger adolescents, such as those born in 2010, were smaller because those persons have had less time for eligibility for inclusion in the NIS-Teen survey. The wider 95% CIs for the more recent birth years should be considered when interpreting and comparing vaccination coverage across different birth years. Finally, a total survey error assessment indicated that NIS-Teen coverage estimates were significantly lower in the 2023 sample compared with the 2022 sample among birth years common to both survey years (*9*,*10*). This analysis might not be able to accurately detect actual changes in survey year coverage from 2022 to 2023 that are within a range of 1–3 percentage points. Possible reasons for the decline in bridging birth year estimates include an early close of the provider record check period in 2023, a change in provider lookup software used by NIS, or a change in reporting by providers. Although data are weighted to account for nonresponse and households without telephones, some bias might remain. Recent total survey error assessments indicated that NIS-Teen estimates might underestimate actual coverage, with the largest underestimation occurring for HPV UTD (−5.2 percentage points) (*10*).

### Implications for Public Health Practice

Health care providers should strongly recommend all routine vaccines and confirm adolescents are fully vaccinated. The VFC program plays a substantial role in this effort by facilitating access to vaccines for eligible families, without financial barriers and by promoting equity in health care access. Parents of adolescents should schedule a well-child visit as the school year begins to verify that adolescents receive all recommended vaccines, such as the HPV vaccine, which prevents 92% of HPV-attributable cancers (*8*). Vaccinations can also be administered through school-based clinics, pharmacies, and back-to-school health events, which can provide expanded opportunities for adolescents to receive recommended vaccines.

## Footnotes

* COVID-19 vaccination is recommended for all persons aged ≥6 months (https://www.cdc.gov/covid/vaccines/stay-up-to-date.html). Estimates of COVID-19 vaccination coverage are available at https://www.cdc.gov/vaccines/imz-managers/coverage/covidvaxview/interactive/children.html.

† Influenza vaccination is recommended for all persons aged ≥6 months. Influenza vaccination coverage estimates are available at https://www.cdc.gov/flu/fluvaxview/index.htm.

§ VFC-eligible adolescents were defined as meeting one of the following criteria: 1) enrolled in Medicaid or Indian Health Service; 2) uninsured; 3) American Indian or Alaska Native; 4) ever received a vaccination at Indian Health Service–operated centers, Tribal health centers, or urban Indian health care facilities.

¶ Persons living in all identified households with mobile telephones were eligible for interview. Sampling weights were adjusted for survey nonresponse, adolescent multiplicity (number of chances for selection), and noncoverage of the survey sampling frame, and were calibrated to known population totals. During 2015–2017, NIS-Teen sampled from a landline frame in addition to a mobile telephone frame; therefore, sampling weights were also adjusted for overlapping samples of mixed telephone users. A description of NIS-Teen single-frame survey methodology and its effect on reported vaccination estimates is available at https://www.cdc.gov/vaccines/imz-managers/coverage/teenvaxview/pubs-presentations/dual-to-single-frame-teen.html.

** Local areas that received federal immunization funds under Section 317 of the Public Health Service Act were sampled separately. Those areas included Chicago, Illinois; New York, New York; Philadelphia County, Pennsylvania; Bexar County, Texas; and Houston, Texas. Three territories were sampled separately in 2023: Guam, Puerto Rico, and U.S. Virgin Islands.

†† The 2023 NIS-Teen sample included 7,857 females and 8,711 males. Adolescents from Guam (131), Puerto Rico (453), and U.S. Virgin Islands (89) were excluded from the national estimates.

§§ Estimates in this report include persons who might have received vaccinations on time or as catch-up. Influenza vaccination coverage data are not included in this report but are available at https://www.cdc.gov/flu/fluvaxview/index.htm.

¶¶ The Council of American Survey Research Organizations household response rate is the product of three other rates: 1) the resolution rate (the proportion of telephone numbers that can be identified as either business or residence), 2) the screening rate (the proportion of qualified households that complete the screening process), and 3) the cooperation rate (the proportion of contacted eligible households for which a completed interview is obtained).

*** Adolescents who received ≥1 non–COVID-19 vaccine dose reported by a provider and those who had received no vaccinations were considered to have adequate provider data. “No vaccinations” indicates that the vaccination status is known because the parent or guardian indicated there were no vaccinations, and the provider returned no immunization history forms or returned them indicating that no vaccinations had been administered.

††† NIS-Teen data during 2015–2023 were combined, and Kaplan-Meier methods were used to calculate cumulative vaccination coverage estimates by age in days, stratified by annual birth year; sample sizes by birth year were 19,931 (2002), 20,085 (2003), 18,908 (2004), 18,242 (2005), 16,564 (2006), 12,633 (2007), 8,346 (2008), 4,990 (2009), and 1,692 (2010). Z-tests were used to compare differences in vaccination coverage by eligibility for the VFC program; differences with p<0.05 were considered statistically significant.

§§§
https://www.cdc.gov/vaccines/imz-managers/nis/downloads/NIS-TEEN-PUF22-DUG.pdf

¶¶¶ 45 C.F.R. part 46.102(l)(2), 21 C.F.R. part 56; 42 U.S.C. Sect. 241(d); 5 U.S.C. Sect. 552a; 44 U.S.C. Sect. 3501 et seq.

**** HPV UTD includes adolescents who received ≥3 doses, and those who received 2 doses when the first HPV vaccine dose was initiated at age <15 years and there was ≥5 months minus 4 days between the first and second dose (https://www.cdc.gov/iis/cdsi/). This update to the HPV vaccination recommendation occurred in December 2016. Some adolescents might have received more than the 2 or 3 recommended HPV vaccine doses.

†††† Tdap vaccination coverage represents coverage with ≥1 Tdap dose at age ≥10 years.

§§§§ Meningococcal conjugate vaccination coverage represents coverage with MenACWY or meningococcal-unknown type vaccine.

¶¶¶¶ HPV vaccination coverage includes receipt of any HPV vaccine and does not distinguish between nine-valent, quadrivalent, or bivalent vaccines. Some adolescents might have received more than the 2 or 3 recommended HPV vaccine doses.

***** MenB vaccination is not routinely recommended for all adolescents. Vaccines are administered to adolescents and young adults aged 16–23 years based on shared clinical decision-making. Coverage estimates for ≥1 and ≥2 MenB doses were calculated among adolescents who were aged 17 years at the time of interview; ≥2 doses of MenB required correct interval between the first and second dose and excluded those with unknown type of meningococcal vaccine.

††††† Vaccination for hepatitis A; hepatitis B; varicella; and measles, mumps, and rubella are considered childhood vaccinations and are recommended for adolescents who are not up to date with these vaccinations. Except as noted, coverage estimates for ≥1 and ≥2 varicella vaccine doses were obtained among adolescents with no history of varicella.
